# Middle Aortic Syndrome Treated by Implantation of an Advanta V12 Large Diameter Stent

**DOI:** 10.5935/abc.20150065

**Published:** 2016-01

**Authors:** Meng-Luen Lee, Ing-Sh Chiu, Albert D Yang

**Affiliations:** 1 Department of Pediatrics, Changhua Christian Children's Hospital, Changhua - Taiwan; 2 Department of Surgery, Changhua Christian Children's Hospital, Changhua - Taiwan; 3 Department of Surgery, National Taiwan University Hospital, Taipei - Taiwan; 4 Medical Imaging, Changhua Christian Children's Hospital, Changhua - Taiwan

**Keywords:** Aortic Diseases, Aorta, Thoracic, Aorta, Abdominal, Stents, Angiography, Balloon.

## Introduction

Middle aortic syndrome (MAS) was coined by Sen et al.^1^ depicting diffuse hypoplasia or stenosis of the distal thoracic aorta and abdominal aorta. Most often MAS is idiopathic^2^; however, it may be associated with a variety of underlying diseases.^2,3^ Systemic hypertension, which is the most common feature of MAS, is usually found incidentally.^2^ Hypertensive encephalopathy was seen in 42% of patients with abdominal aortic coarctation, 45% of whom died before reaching 34 years of age.^4^ Thus, early recognition of hypertensive encephalopathy is crucial for patient survival. We report of a 12-year-old girl presenting with hypertensive encephalopathy as a harbinger of idiopathic MAS, which manifested as an isolated long-segment mid-thoracic aortic coarctation, and was successfully treated by endovascular implantation of an Advanta V12 Large Diameter Stent (AVLDS, Atrium, Hudson, NH, USA).

## Case report

**Figure 1:**
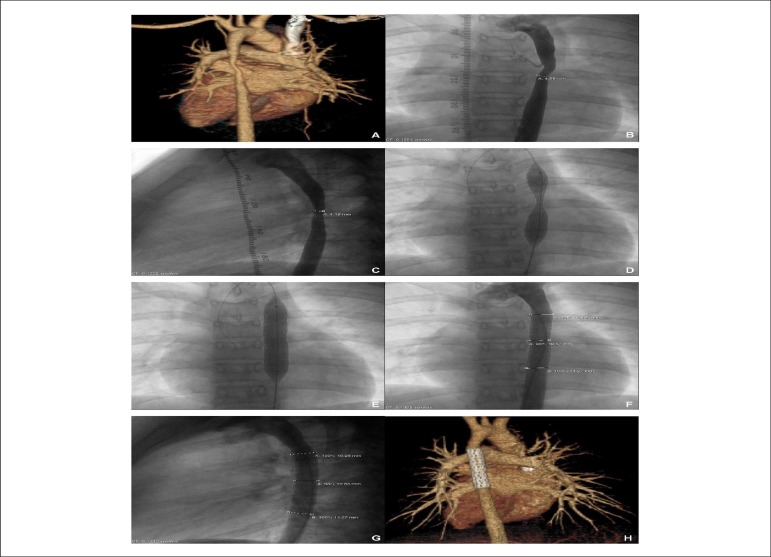
Computed tomography (1A), frontal thoracic aortography (1B), and lateral thoracic aortography (1C) showed an isolated long-segment mid-thoracic aortic coarctation. A dumbbell morphology (1D) was visualized before full expansion of an Advanta V12 Large Diameter Stent (AVLDS). After implantation, repeated thoracic aortography (1E) showed full AVLDS dilatation. Follow-up thoracic aortography (1F and 1G) and computed tomography (1H) showed in situ AVLDS anchoring to the aortic wall effectively and covering the full length of the coarctation sufficiently.

A 12-year-old girl, who had been treated with anti-epileptic agents for intractable seizures for two months, was referred to our hospital with a chief complaint of hypertension. According to her parents, she initially presented with symptoms of hypertensive encephalopathy before experiencing an attack of generalized tonic-clonic seizure, including headache, dizziness, and mental change. At referral, the blood pressures of the upper and lower limbs were 200/126 mmHg and 113/90 mmHg, respectively, and the radial pulses were bounding. Electrocardiography showed left ventricular hypertrophy by voltage. Echocardiography, erythrocyte sedimentation rate, blood urea nitrogen, creatinine, supine renin activity, supine renin level, and supine aldosterone level were all within normal limits. Computed tomography (Figure 1 A ) and angiography (Figure 1 B and 1C) identified the cause to be MAS, manifesting as an isolated long-segment mid-thoracic coarctation. There was a pressure gradient of 74 mmHg (187 - 113 mmHg) across the coarctation. Intervention was declined, and antihypertensive agent was prescribed. Hypertension of the upper limbs lingered. The patient had headache and dizziness during the flare-ups of hypertension (190-200 mmHg). Endovascular intervention was performed one year later. The pressure gradient was 86 mmHg (193 - 107 mmHg). Stent implantation (SI) was declined because of the expensive charge. Instead, we performed balloon angioplasty (BA) using two sets of Wanda Balloon Catheter (8.0 mm and 10.0 mm x 40 mm; Boston Scientific, Galway, Ireland). The lesion was dilated to 6.76-6.98 mm. Stenosis ratio improved from 47%-51% to 24%-36%, and the pressure gradient dropped from 86 mmHg to 46 mmHg (158 - 112 mmHg). However, the patient was haunted by headache and dizziness at the hypertensive flare-ups (180/100 mmHg), despite treatment with antihypertensive agent.

We recommended that the treatment gap between the neurological presentations of hypertensive encephalopathy and the interventional procedure of SI for mid-thoracic aortic coarctation should not be intentionally delayed or postponed, notwithstanding her neurological symptoms improving or becoming refractory. Finally, SI was accepted by her parents and performed one year later. To achieve a maximal diameter of the target lesion in this growing adolescent, we chose a 12 mm x 61 mm AVLDS to treat this lesion (Figure 1 D and 1E). AVLDS was distended gradationally by increasing the balloon pressure from a nominal pressure of 8 atm to a burst pressure of 12 atm. The outer diameter of the expanded balloon of this chosen stent (12.3 mm at 8 atm; 12.6 mm at 12 atm) is estimated to be at least 2.5 times the diameter of the lesion (4.18-4.88 mm). It appeared useful to anchor this device effectively to the aortic wall to prevent it from slipping. After SI, the lesion was dilated from 6.38-6.45 mm to 10.57-1 0.68 mm (Figure 1 F and 1G). The pressure gradient dropped from 64 mmHg to 3 mmHg. Stenosis ratio improved from 57%-62% to 4%-6%, by comparing the diameters of the coarctation (before SI: 4.18-4.88 mm; after SI: 10.57-10.68 mm) with those of the normal distal thoracic aorta (11.07-11.27 mm). Follow-up computed tomography showed AVLDS *in situ* covering the full length of the mid-thoracic coarctation (Figure 1 H). The patient was free of the sequelae of hypertensive encephalopathy over the 36-month follow-up.

## Discussion

BA has been advocated as an effective modality for relieving stenosis of the aorta in children.^5^ SI has been advised as an alternative if facing flow-limiting dissection, ineffective BA, and recurrent restenosis after suboptimal BA.^6^ Thus, BA should not be considered as a sole therapy in patients with MAS, especially in those with the abdominal aortic coarctation near or across the orifices of both renal arteries (with or without a company of renal artery stenosis). In such a scenario, BA may be plagued with bad results due to the obliteration of renal arteries. Although kissing BA may serve as an alternative, surgery has a pivotal role in correcting such anomalies with simultaneous long-segment abdominal aortic coarctation and orifice renal artery stenosis at the junctions. Brzezinska-Rajszys reported of five children presenting with long-segment stenosis of the mid/lower thoracic and upper abdominal aorta due to MAS, which was palliated by the implantation of Palmaz stent prior to surgery.^7^ The Palmaz stent requires a delivery system with smaller profiles (8-11 Fr) than the profile of the Cheatham-Platinum (CP) stent (10-16 Fr). The issue with the Palmaz stent is that it fails to keep up with the somatic growth of the smaller pediatric patients. On the other hand, the CP stent is too large a device for smaller children and its use is limited only to adults. To overcome this treatment dilemma, a satisfactory solution is the emerging technique of AVLDS, which is a balloon-expandable stent made of 316L stainless steel and encapsulated with expandable graft material made of polytetrafluoroethylene. AVLDS can be deployed through a smaller delivery system (9-11 Fr) than that of the CP stent and can be dilated up to 22 mm approximating the grown-up size of the abdominal aorta in adults. These attributes render AVLDS an optimal choice for smaller children with potential somatic growth. AVLDS has been used to relieve various types of cardiovascular obstruction in 18 patients with good immediate results,^8^ and achieved successful short-term results in 25 patients with coarctation of the aortic arch;^9^ moreover, it was used once in a 13-year-old boy with a similar long-segment thoraco-abdominal aortic coarctation due to idiopathic MAS, as in our case, providing significant reduction in blood pressure.^10^ The clinical caveats of the implantation of an AVLDS are two-fold. First, it is important that the AVLDS should be chosen beforehand so that both ends of the stent reach beyond the area of any post-stenotic dilatation, aneurysm, or dissection.^9^ Second, to overcome the intrinsic elastic recoil of polytetrafluoroethylene and to prevent the stent from slipping, it is important to anchor the AVLDS to the wall using an initial balloon up to 2.5 times the diameter of the coarctation.^9^

We also recommend that the treatment gap between the neurologic presentations of hypertensive encephalopathy and the interventional procedure of SI for such a long-segment mid-thoracic aortic coarctation should not be intentionally delayed or postponed, despite the fact that patient's neurological symptoms got improved or remained refractory.

## Conclusion

Hypertensive encephalopathy can be a harbinger of idiopathic MAS. AVLDS is an ideal modality to treat an isolated long-segment mid-thoracic coarctation in an adolescent with potential somatic growth.
